# The roles of multi-component interventions in reducing mistreatment of women and enhancing respectful maternity care: a systematic review

**DOI:** 10.1186/s12884-023-05640-3

**Published:** 2023-05-01

**Authors:** Habtamu Kasaye, Annabel Sheehy, Vanessa Scarf, Kathleen Baird

**Affiliations:** 1 Collective for Midwifery, Child and Family Health, Faculty of Health, University of Technology Sydney, Sydney, Australia; 2 Department of Midwifery, Institute of Health Sciences, Wollega University, Nekemte, Ethiopia

**Keywords:** Mistreatment of women, Respectful maternity care, Multi-component interventions

## Abstract

**Background:**

Despite recognition of the adverse impacts of the mistreatment of women during pregnancy, labour and birth, there remains limited evidence on interventions that could reduce mistreatment and build a culture of respectful maternity care (RMC) in health facilities. The sustainability of effective individual interventions and their adaptability to various global contexts remain uncertain. In this systematic review, we aimed to synthesise the best available evidence that has been shown to be effective in reducing the mistreatment of women and/or enhancing RMC during women’s maternity care in health facilities.

**Methods:**

We searched the online databases PubMed, CINAHL, EBSCO Nursing/Academic Edition, Embase, African Journals Online (AJOL), Scopus, Web of Science, and grey literature using predetermined search strategies. We included cluster randomized controlled trials (RCTs) and pre-and-post observational studies and appraised them using JBI critical appraisal checklists. The findings were synthesised narratively without conducting a meta-analysis. The certainty of evidence was assessed using GRADE criteria.

**Results:**

From the 1493 identified records, 11 studies from six sub-Sahara African countries and one study from India were included: three cluster RCTs and nine pre- and post-studies. We identified diverse interventions implemented via various approaches including individual health care providers, health systems, and policy amendments. Moderate certainty evidence from two cluster RCTs and four pre- and post-studies suggests that multi-component interventions can reduce the odds of mistreatment that women may experience in health facilities, with odds of reduction ranging from 18 per cent to 66 per cent. Similarly, women’s perceptions of maternity care as respectful increased in moderate certainty evidence from two cluster RCTs and five pre- and post-studies with reported increases ranging from 5 per cent to 50 per cent.

**Conclusions:**

Multi-component interventions that address attitudes and behaviors of health care providers, motivate staff, engage the local community, and alleviate health facility and system constraints have been found to effectively reduce mistreatment of women and/or increase respectful maternity care. Such interventions which go beyond a single focus like staff training appear to be more likely to bring about change. Therefore, future interventions should consider diverse approaches that incorporate these components to improve maternal care.

**Supplementary Information:**

The online version contains supplementary material available at 10.1186/s12884-023-05640-3.

## Introduction

Pregnancy, childbirth, and the early parenting period are remarkable events in a woman's life, with women encountering a range of experiences such as joyful, positive transitions through to periods of trauma and vulnerability [1, 2]. To enhance positive outcomes, health systems and health care providers (HCPs) must ensure high-quality, equitable, evidence-based, and respectful maternity care (RMC) for all women [3]. The absence or deficiency of RMC manifesting as the mistreatment of women diminishes the quality and efficacy of maternity care across all cultures [4]. Mistreatment is perceived when the provision of maternity care is perceived by women to be disrespectful, abusive, neglectful, or undignified [4, 5]. The prevalence of women experiencing at least one form of mistreatment ranges from 17.3 per cent in the United States of America to 44 per cent in sub-Saharan African (SSA) [6–10]. Despite the contextual variation between settings and across countries, it is important to note that mistreatment is any humiliating encounter that women experience in a health facility [4, 5]. It is important to note that perceived mistreatment can have long-lasting negative impacts on a woman’s dignity and self-esteem and negatively impact mothering and decisions for future childbearing [11–13].

Mounting evidence demonstrates varied adverse impact of the mistreatment of women and its deterrence of women's utilisation of maternal health care. Experiences of mistreatment result in dissatisfaction of received care and lower confidence in maternity care, and curtail subsequent health-seeking behaviours [14–17]. In particular, perceptions of distrust of health care facilities by women in low- and middle-income countries have been shown to override socio-cultural beliefs about the importance of accessing maternity care [18]. Even though the impact of mistreatment is known, there is limited specificity of information for developing ameliorative strategies to build a culture of RMC in maternity services and prevent cultures of mistreatment and clinical encounters within which women are disrespected, abused, neglected, and/or degraded.

Organisations such as the White Ribbon Alliance and the World Health Organization (WHO) highlight the urgent requirement for all women to have access to maternity care that is safe and respectful [19–21], however, there is a lack of practical and sustainable interventions which enhance respectful care and reduce mistreatment of women. Studies from low-income countries suggest that training interventions aimed at transforming attitudes, values and behaviours of health care providers can bring about some positive effects in maternity care [22, 23]. Similarly, the mobilisation of communities to demand RMC, as well as dispute resolution strategies for women who have experienced mistreatment can result in changes which may minimise abusive care [22, 23]. In a systematic review comprising of five African studies, Downe et al. [24] concluded that policy interventions could generate changes to minimise the mistreatment of women during maternity care. However, it is uncertain from this review as to the degree of effectiveness of specific components of the interventions, and their sustainability and adaptability to varying global contexts, especially communities with resource limitations [24]. A recent mixed-method review which focused on educational interventions for health care providers was shown to understanding of RMC by staff, yet Dhakal et al. [25] concluded that it was not evident whether the included interventions impacted women's perceptions of actual mistreatment.

Reducing mistreatment is complicated and requires strategies that focus beyond health care providers' attitudes, behaviours and actions [26]. At the institutional and policy level, the resource constraints and staffing deficits of health facilities and health systems significantly worsen the experiences of women and families [4, 27]. Research examining the phenomenon of mistreatment of women from various viewpoints is therefore required. Identifying specific components of RMC interventions that successfully and sustainably reduce women's mistreatment is necessary. This systematic review aimed to synthesise the best available evidence on such interventions which utilise differing approaches to address the issue of mistreatment. The effectiveness of these interventions in reducing the mistreatment of women and/or enhancing respectful maternity care in health facilities was also examined.

## Methods

A systematic review was performed in accordance with the Preferred Reporting Items for Systematic Reviews and Meta-Analysis-2020 (PRISMA-2020) guideline [28], as shown in supporting file S1 Table, and also followed the systematic review protocol registered in the International Prospective Register of Systematic Reviews (PROSPERO) with registration number— CRD42021287049.

### Eligibility criteria

As described below, the studies were selected according to the PICOS (participants/population, intervention, comparisons, outcomes, and study designs).

#### Population

The study population was women receiving maternity care from health facilities and maternal health care providers. Although respectful care and mistreatment-free services are equally important for other health care recipients from health facilities, our focus in this review is on services provided to women during maternity care. Studies that evaluated outcomes reported by women who received maternal health care from health facilities or health care providers were included.

#### Interventions

Studies that evaluated the effectiveness of RMC interventions at any level including the community, health facility, and individual health care providers in reducing mistreatment of women and/or enhancing respectful maternity care were included. The interventions were included in the review based on the outcome that they intended to evaluate. Hence, interventions which targeted various levels and components to change health care providers’ attitudes and behaviours, health facilities and system failures, and change how women react to the abusive and disrespectful care were included. We excluded interventions that did not primarily focus on reducing mistreatment of women and/or enhancing respectful care, but instead aimed exclusively at achieving unrelated outcomes such as increasing service utilisation or decreasing specific interventions of childbearing.

#### Comparator

The effectiveness of the RMC interventions was compared with the standard usual routine care which existed prior to the implementation of the RMC interventions.

#### Outcomes

The primary outcome sought in this systematic review was whether the level of mistreatment that women experienced could be reduced secondary to the implementation of the interventions. Mistreatment was defined as explicit experiences in childbirth, such as verbal, sexual, and physical abuse, neglect, stigma and discrimination, poor communication, and/or other forms of mistreatment related to health care providers or health care facilities, as described in Bohren et al.'s [4] and Bowser and Hill's [5] global reviews. Articles which evaluated the occurrence of any of these disrespectful and abusive care were included. Hence, studies which included evaluations of the intervention, either as observations of the levels of women's mistreatment between the two groups or those that included the self-reported experiences of women themselves, were also included. Overall mistreatment of women could be measured as occurrences in any form or the proportion of occurrences of each component, such as physical abuse, verbal abuse (also known as non-dignified care, non-consented care, nonconfidential care, discrimination, neglect, or abandonment of care), and detention in health facilities. It could also extend to poor rapport/communication between women and health care providers.

Another outcome of interest was respectful maternity care, as reported by women or observed by the investigators. Respectful maternity care is a woman-centred maternity care that is organised for, and provided to, all women by upholding their privacy, confidentiality, and dignity, protecting women from harm and mistreatment through the provision of continuous support and enabling active decision making by women throughout their pregnancy, childbirth and during postnatal care [29]. Respectful maternity care improvement is measured as changes in the domains of respectful maternity care which include being free from harm and mistreatment, the protection of dignity, privacy and confidentiality, informed consent, and respect for their preferences among others as suggested by Shakibazadeh et al. [30]

### Types of studies

Cluster RCTs and pre-and-post interventional studies with and without control groups were included in the review.

### Database selection and search strategy

The search was conducted from 10 November 2021 through December 13, 2021, to retrieve both published and unpublished studies. An initial search of PubMed and CINAHL databases was performed based on the eligibility criteria mentioned under eligibility criteria. After analysing the text and terminology used in potential studies identified from PubMed and CINAHL databases, a second search was undertaken using all identified keywords and index terms performed in EBSCO Nursing/Academic Edition, Ovid Embase, African Journals Online (AJOL), Scopus, Web of Science, and Google Scholar.

Manual searches of the reference lists of all identified papers and previous systematic review papers were performed to identify studies cited within the selected papers. Searches for unpublished studies in ProQuest's dissertation and thesis database, grey literature from search engines such as Google, WHO Global Health Library websites, White Ribbon Alliance, and the International Confederation of Midwives were also performed. The keywords used in the initial search included *'mistreatment'*, 'respectful maternity care*'*, *'maternity care'*, and *'*interventions*'*. The details of the database search strategies are provided in the supporting file S2 Table.

### Data management and study selection

The results from all database searches were exported to Endnote version X9 [31] for storage and management. The results were labelled in Endnote as either originating from the database or manually searched. Bibliographies captured in Endnote were then exported to the Covidence Systematic Review software (Veritas Health Innovation, Melbourne, Australia; available at www.covidence.org) for the screening and identification of relevant studies, and duplicates were removed.

The remaining titles and abstracts were assessed for relevance to the eligibility criteria independently by HK and VS. Following title and abstract screening, papers deemed relevant to the review were then reviewed in full-text form. Further review by KB and AS resolved any discrepancies within the initial review process.

### Data collection and analysis

#### Assessment of methodological quality

Papers selected for retrieval were assessed independently by HK and AS to ensure methodological quality using standardised critical appraisal instruments from the JBI System for the Unified Management, Assessment, and Review of Information (JBI SUMARI; JBI, Adelaide, Australia) for quasi-experimental (for pre-and-post non-randomised interventional studies) and RCTs [32]. Differences in opinion between the two reviewers were resolved through discussion, and a consensus was reached with the involvement of KB and VS. No studies were excluded based on the results of the critical appraisal.

#### Data collection and synthesis

Data were extracted from the included studies using a checklist developed for this purpose by HK and VS, and agreement reached by consensus by re-examination of the queried studies by AS and KB. Data were only used when there was consensus from all the authors. The extracted data included study authors, year of publication, study country and setting, study participants, sample sizes, recruitment methods, study design, interventions, reported outcomes, measurement means, and effect measures as detailed in Table 1.

**Table 1:** Characteristics of included studies

Lead author (citation) Country	Characteristics
Abuya et al. [23] Kenya	**♢ Design**: multicenter pre-and post-study without a control group **♢ Participants**: Women aged 15 to 45 years were surveyed for their experiences within 24–48 h of birth at discharge **♢ Sample**: **1,369** (641 women at baseline and 728 women at endline) **♢ Intervention**: Multi-component intervention implemented at facility, community, and policy levels in the *Hashima* project from June 2011 – Feb 2014 ○ **Community level** - Community workshops- civic education for the community on the right to sexual and reproductive health, sensitization meetings with the community members to demand respectful care - Counseling community members who experienced disrespect and abuse by the counselors in the facility ○ **Facility level** - Providing training for health care providers (HCPs) that aimed at enhancing the protection of clients' and providers' rights through improving quality of care - Caring for carers- counselling services for HCPs to assist them cope with high workload, critical incidents, and trauma - Monitoring D&A- through facilitating incident reporting mechanisms - Mentorship- in-service role-modeling the champion provider behavior as routine continuous professional education - Maternity open day- trust-building session prepared in health facility through explaining procedures in the maternity ward for the invited members of the local communities ○ **Policy level** - Continuous policy dialogue with government, civil society, and professional knowledge networks **♢ Outcome**: women reported and observed experiences of disrespect and abuse **♢ Measurement:** measured as dichotomous variable, percentage of women responding to six questions asking whether they were disrespected or humiliated at least for one form of categories of D&A **♢ Evaluation of effect:** multivariate logistic generalized linear mixed models (GLMM) were used for both observational and exit interview data and reported as both adjusted and crude odds ratios (OR) **• Observation**: lack of privacy and physical aggression were reduced from baseline while non-consented care and sharing bed were increased from the baseline level **• Survey with women**: overall D&A was reduced from baseline level 129(20.1%) to 96(13.2% (– adjusted OR: 0.55 (0.40 – 0.75) **•** Physical abuse, verbal abuse, violation of confidentiality and detention were also significantly declined from their baseline levels Reliability and validity of the survey tool were not shown
Afulani et al. [33] Ghana	**♢ Design**: Pre- and post-intervention study without control groups was conducted in five high volume childbirth health facilities (one hospital and 4 health centers) **♢ Participants**: women aged 15–49 years who give birth in preceding 8 weeks in study health facilities were approached at discharge from facilities **♢ Sample**: 215 women at baseline (March–April 2017) and 318 at endline (November 2017) **♢ Intervention**: Two days *integrated simulation‐based training* was provided for 43 health care providers including midwives, medical doctors, anesthetists, and nurses in two rounds. The content of the training includes: - Five simulation scenarios - Skills capturing session in identified seven areas of focus **♢ Outcome:** RMC as reported by women **♢ Measurement**: RMC measured as a continuous variable of score of person-centered maternity care scale (PCMC) structured into 4-point Likert scale from 24 items and then converted to 100 **♢ Evaluation of the effect:** RMC, average PCMC increased by 43% from 50 (baseline score) to 72 (endline score); it was also reported as increased in linear regression coefficient (18 points than the baseline score, (β = 17.6; 95% CI = 15.6‐19.6) • Dignity and respect, communication and autonomy and supportive care were subclass indictors showed increment **♢ ***Used a tool validated in Kenya and India with high content, construct, and criterion validity with a good reliability*
Asefa et al. [34] Ethiopia	**♢ Design**: Pre- and post-study without a control group conducted between December 2017 and September 2018 in three Hospitals in Southern Ethiopia **♢ Participants**: women who gave birth in study facilities took part in the study at discharge **♢ Sample**: 388 women were surveyed- 190 before intervention and 198 after intervention **♢ Intervention**: Respectful Maternity care training- the contents and intensity of the interventions implemented include: ○ **Facility level** - Respectful maternity care training for health care providers- three-day workshops for 64 HCPs in two rounds - Five wall posters: four in English and one in Amharic posted in labor ward to be used as on job aids incorporating universal rights of childbearing women developed by White Ribbon Alliances and infographics prepared by WHO - Supportive supervisions: two round quality improvement post-training supportive supervisions were conducted through developing action plans for the standard-based identified gaps **♢ Comparator**: usual care before the implementation of the intervention **♢ Outcome**: women's experiences of mistreatments **♢ Measurement**: measured as number of mistreatment categories women experienced using 25 items originating from six categories of mistreatment **♢ Evaluation of the effect**: the effect of the intervention was evaluated using a multilevel mixed-effects Poisson regression model • Adjusted exponentiated regression coefficient = 0.82, 95% CI 0.74 to 0.91
Asefa et al. [35] Ethiopia	**♢ Design:** Pre- and post-intervention study without a control group conducted between April and May 2018 **♢ Participants:** health care providers who provide labor and childbirth and received intervention participated in the survey **♢ Sample:** 64 HCPs responded to pre intervention survey and all of them participated in post intervention survey **♢ Intervention:—Facility level as described above in Asefa et al.** [34]**,** - Three days training delivered as presentations, role play, demonstrations, case studies, individual readings, videos, and a hospital visit - Contents of training- overview of maternal health in Ethiopia, human rights, and law in the context of reproductive health, RMC rights and standards, professional ethics, and continuous quality improvement **♢ Outcome**: HCPs perceptions of RMC **♢ Measurement**: perceptions of RMC was measured using eight domains and classified it as positive and negative perceptions **♢ Evaluation of the effect:** an exact McNemar's test was performed to analyze pre-post differences in participants' perceptions of RMC - Proportion of perceiving RMC domains positively was 21.9% before the training, and 35.9% after the training (*p* = 0.08)
Brown et al. [36] South Africa	**♢ Design:** a pilot cluster RCT study conducted at 10 hospitals (five randomly allocated to receive educational intervention to promote childbirth companion) **♢ Participants:** Postnatal women who received labor and birth care in study hospitals **♢ Sample:** 2090 survey before intervention (October 1998) and 2058 exit interviews carried out after intervention (December 1999) **♢ Intervention**: Childbirth companion promotion- a multidimensional educational intervention delivered as interactive workshop for HCPs, banners and posters at labor ward, brochures and video program promoting birth companion **♢ Comparator**- the five control hospitals received an unrelated evidence-based intervention to promote the external cephalic version (ECV) **♢ Outcome:** birth companion and indicators of mistreatment (described as inhuman care in study- being shouted at, being slapped, or struck, being left alone) **♢ Measurement:** self-reported by women whether they were allowed a companion, and experiencing inhumane care mentioned **♢ Evaluation of the effect:** Used non-parametric test (Mann Whitney U test) and did not report the effect size, only reported it as there were no significant effect
Kujawski et al. [22] Tanzania	**♢ Design**: cluster RCTs **♢ Participants**: women aged 15 and above and gave birth in study facilities were participated in exit interview **♢ Sample**: 3068 women were included (Baseline**:** 1388 (744 from control hospital and 644 from intervention hospital) and (**Endline:** 1680 (769 from control hospital and 1001 from an intervention hospital) **♢ Intervention**: *Staha* intervention comprising two components was implemented over two years in Korogwe District, Tanzania and compared with Muheza District as: ○ **Community level** **- Client service charter-** community and health facility stakeholders adapted client service charter. The charter was issued to the communities and posted in health facilities found within the intervention district ○ **Facility level:** **- Quality improvement program-** following the adaptation of the client service charter for six-month, quality improvement activities that activated charter content were performed to address disrespectful and abusive care **♢ Comparator-** compared to women gave birth in health facilities without any intervention (usual care) and to the practices existing before interventions **♢ Outcome**: women's self-reported experiences of any form of D&A **♢ Measurement**: labeled as experienced D&A if women reported at least one of 14 questions during labor and birth based on the Bowser and Hills' categories **♢ Evaluation of effect:** adjusted logistic regression difference in difference model between baseline and endline on a total of 2983 eligible survey results • 66% reduced odds of a woman experiencing D&A (adjusted OR: 0.34, 95% CI: 0.21–0.58, *p* < 0.0001) • The biggest reductions were for physical abuse (aOR: 0.22, 95% CI: 0.05–0.97, *p* = 0.045) and neglect (aOR: 0.36, 95% CI: 0.19–0.71, *p* = 0.003) **♢** *The validity and reliability of the survey tool was not reported*
Mihret et al. [37] Ethiopia	**♢ Design:** Pre-and-post single center without a control group interventional study from November 2018 to May 2019 **♢ Participants:** women who received antenatal care and gave childbirth in study hospital were surveyed for their experiences before and after intervention **♢ Sample: 738 (**369 at baseline and 369 at endline/after intervention) **♢ Intervention:** RMC and monitoring and evaluation training for HCPs and managers, setting up waiting room, availing resources for ensuring privacy (curtains), essential, drugs written guideline and protocol, recognizing best performing staff and continuous supportive supervision by quality improvement team **♢ Comparator-** pre intervention services **♢ Outcome:** proportion of disrespect and abuse among pregnant women who received ANC and labor and birth in health facility **♢ Measurement:** The D&A was identified as any form of abusive care using 24 Yes/No questions based on Bohser and Hill's categories of D&A **♢ Evaluation of the effect:** significance of the intervention was checked using independent t- test, reported as significant **♢** Overall D&A before intervention was 71.8 and 15.9% after intervention
Montagu et al. [38] India	**♢ Design:** cluster RCT was conducted at three primary health centers and six community health centers of Unnao and Kanpur Districts of Uttar Pradesh state in India **♢ Participants:** women aged 18–49 and gave childbirth within last seven days in participating health facilities **♢ Sample:** 570 (285 at each group) women at baseline from September 2016 to March 2017 and 600 (300 at each group) women at endline from May to December 2018 participated in surveys **♢ Intervention:** establishing quality improvement team and participation in Improvement Collaborative workshops to work towards the improvement of person-centred maternity care through a plan-do-study-act (PDSA) **♢ *Comparators:*** usual care at non-interventional control health facilities **♢ Outcome:** Person-centered maternity care (PCMC) that includes RMC domains (dignity and respect; communication and autonomy; and supportive care.) **♢ Measurement:** measured using 23 validated survey items scaled to 100-point scale, highest score indicating better PCMC/RMC care **♢ Evaluation of the effect:** from baseline to endline, the adjusted mean PCMC score of the intervention group increased 22.9 points (95%CI: 20.9, 25.0) **♢ After the intervention-** PCMC mean score for intervention group was 97.13 with SD of (2.91) and in a control group mean score 63.42 with SD of (11.44)
Oosthuizen et al. [39] South Africa	**♢ Design:** Pre- and post-pilot interventional study in Tshwane health district in 10 midwife-led obstetric units (MOUs) of South Africa. Five MOUs purposively selected to be part of the intervention while the remaining five were treated as a control group **♢ Participants:** women who had given child in MOUs and returned for postnatal care from 3 days to six weeks **♢ Sample:** 653 women at baseline from February to April 2016 and 679 at endline survey from October 2016 to March 2017 **♢ Intervention:** CLEVER package- that includes awareness creation of women's experiences for strengthening health system with MOUs' participants, intensive behavioral change activities for 3-months and six-month support **♢ Comparators:** usual care before intervention and care in control groups **♢ Outcome:** RMC and satisfaction **♢ Measurement:** validated survey tools were used to measure RMC by a question that asked to rate how women feel they were respected and the other rate their level of satisfaction **♢ Evaluation of the effect:** the percentage of RMC changed from 38.1% at baseline to 74.5% endline, and satisfaction from 47% to 73.6% ○ RMC (aOR = 4.33) and satisfaction (aOR = 4.04) raised four times at endline as compared to baseline in the interventional groups (*P*-values < 0.0001) RMC (aOR = 1.14) and satisfaction (aOR = 1.20) raised at endline as intervention compared to control groups (*P*-values < 0.0001) **♢** As compared to the control groups, endline RMC was 71.6% for controls and 74.5% for interventional groups, while endline satisfaction was 71.1 for control groups and 73.6% for interventional groups
Ratcliffe et al. [40] Tanzania	**♢ Design:** Pre- and post-intervention study between January 2013 and December 2014 at large, urban regional referral hospital in Dar es Salaam, Tanzania **♢ Participants:** women who gave birth at the facility during four to six weeks post-delivery in the woman's home **♢ Sample:** 70 women at baseline and 149 women after intervention **♢ Interventions:** two discrete interventions were implemented: ○ **Health facility level** - Open Birth Days (OBD), a birth preparedness and antenatal care education program for women while they are in third trimester pregnancy - Workshop with HCPs on respectful maternity care aimed at examining practice with respect to professional code of conduct, clients' preferences and discussion on barriers that prevent provision of RMC **♢ Comparator:** service existing in the facility before the implementation of the intervention **♢ Outcome:** women's experiences of respectful care and satisfaction with care **♢ Measurement:** RMC measured as the perception of the women rating how health care providers were respectful (five Likert scale question) **♢ Evaluation of the effect:** measured as the change in percentage of the RMC perception and very satisfied - Perception of respectful care: 22.8% of women rated the respect shown to them by providers as "excellent" compared to none at baseline - Satisfaction: 75.8% of women reported being very satisfied with their birth experience compared to only 12.9% at baseline
Ratcliffe et al. [41] Tanzania	**♢ Design through intervention-** as described above in Ratcliffe et al. [40] **♢ Outcome:** experiences of disrespect and abuse as reported by women **♢ Measurement:** D&A was measured using on the items developed based on the Bowser and Hills' categories of D&A **♢ Evaluation of outcome:** evaluated as percentage difference between occurrences of individual categories of D&A and any form of abusive care as dichotomous variable: **♢ Any form of D&A:** 70% at baseline and 18% at endline
Umbeli et al. [42] Sudan	**♢ Design:** Pre- and post-intervention study was conducted in Omdurman maternity hospital, Sudan **♢ Participants:** women who gave childbirth in study facility were surveyed **♢ Sample:** a total of 4469 women were surveyed (2000 before and 2469 after) training **♢ Intervention:** training HCPs on communication skills, support during childbirth, providing information and empathy **♢ Comparators:** usual care before intervention **♢ Outcome: respectful care and satisfaction level** **♢ Measurement:** RMC measured as a women's perceptions of friendly and respectful care **♢ Evaluation of the effect:** difference between women's opinion about health care providers' behavior in labor ward ○ The proportion of HCPs who were supportive, friendly, and respectful was 1793 (89.7%) before training and 2338 (94.7%) after training **♢** Proportion of women received information on onset of labor from 76.8% to 96.8%, requested investigations from 54.9% to 94.5%, condition of the fetus from 15.3% to 92.1%, progress of labor from 9.9% to 89.9%, expected duration of labor from 8.9% to 95.0%, examination and procedure to be done from 7.5% to 57.9% from baseline level to the endline

Although statistical pooling via a meta-analysis of the effects of the interventions was our preferred and planned method of synthesis per protocol, a meta-analysis of effect estimates could not be achieved due to extreme clinical and methodological heterogeneity between the included studies. The interventions in the included studies were highly varied in terms of mode of action: including various educational/training packages for health care providers, diverse community-level interventions, and policy-and health facility-level quality improvement strategies. The characteristics of the included studies are summarised in a table utilising the PICO format. This enabled the comparison of studies, including settings, population of interest, interventions being implemented, and outcomes evaluated. The outcomes of the studies were analysed for their effectiveness and then synthesised as a descriptive narrative, utilising the format of texts as recommended when there is synthesis of findings without meta-analysis [43]. The effect measures from each study were extracted and interpreted as reported by primary authors as relative or absolute risk measures— including adjusted odds ratios and relative risks for dichotomous data while mean difference between groups in studies measured outcome as continuous variables.

### Assessing certainty in the findings

The Grading of Recommendations, Assessment, Development, and Evaluation (GRADE) approach for systematic reviews was used to rate the certainty of the evidence for mistreatment of women, sub-categories of mistreatment of women (physical abuse, verbal abuse, non-confidential care, non-consented care, and violation of privacy), and respectful maternity care. The final grading of the certainty of the evidence was agreed by consensus of all reviewers after initially graded independently by HK and VS. A summary of the findings is presented.

## Results

### Search results and study selections

**Fig. 1:**
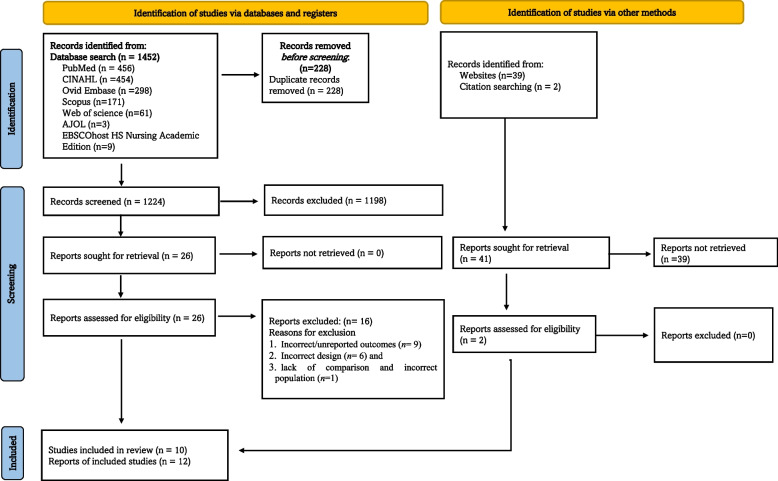
Study selection flow diagram

In the initial systematic search, 1493 studies were retrieved using electronic databases and other methods. After removing duplicates, 1265 studies were screened by examining their titles and abstracts, of which 28 were retrieved for full-text assessment. Sixteen of these studies were excluded due to incorrect or unreported outcomes (*n* = 9), incorrect design (*n* = 6), lack of comparison and incorrect population (*n* = 1), as depicted in Fig. 1.

### Characteristics of included studies

Owing to the wide range of methodological inadequacies of the studies, all selected reports were included in the analysis for pragmatic reasons, which were relevant to the aim of the review and comprised the highest available methodological rigor of all assessed studies. Three of the 12 included studies were RCTs [22, 36, 38], and the remaining nine were pre-and-post/ quasi experimental studies [23, 33–35, 37, 39–42]. Out of the 12 studies included, it is worth noting that regardless of no criteria used to exclude studies based on their geographical region, eleven studies were conducted in sub-Saharan Africa (specifically, in Ethiopia, Tanzania, South Africa, Ghana, Kenya, and Sudan) and one study was conducted in India.

A total of 16,834 women and 64 health care providers participated in the studies, and the numbers in the intervention and comparison groups (control or pre-intervention) ranged from 219 to 4469 participants (mean 945, median1035) with an age of 15 years of age and above. In addition to identifying the effects of interventions during birth, Mihret et al. [37] also assessed the effects of interventions on disrespect and abuse during antenatal care. Exit interviews following childbirth or antenatal care and community follow-up surveys were the main approaches for data collection. Asefa et al. [35] identified the effects of interventions from health care providers' perspectives. The overall characteristics of the included studies are presented in Table 1.

### The methodological quality of included studies

A JBI critical appraisal checklist was used to assess the methodological quality of each study. Summaries of these assessments are presented in Tables 2 and 3. All quasi-experimental studies scored positive ('Yes') for more than half of the appraisal domains, ranging from five to seven of nine quality domains. In four out of nine quasi-experimental studies [23, 33, 37, 39], the participants in the comparison groups were not similar. In contrast, all studies identified causes (interventions) and effects (mistreatment and/or respectful maternity care). Only Oosthuizen et al. [39] included a parallel control group before and after the intervention. The remaining quasi-experimental studies used the survey findings conducted before the interventions were implemented as comparison groups.

**Table 2:** Critical appraisal results for the quasi-experimental studies included in the review

Studies	Critical appraisal questions									Score
	Q1	Q2	Q3	Q4	Q5	Q6	Q7	Q8	Q9	
**Abuya et al. **[23]	Y	N	Y	N	N	NA	Y	Y	Y	6
**Afulani et al. **[33]	Y	N	Y	N	N	NA	Y	Y	Y	6
**Asefa et al. **[35]	Y	Y	Y	N	N	Y	Y	N	N	5
**Asefa et al. **[34]	Y	Y	Y	N	N	NA	Y	Y	Y	7
**Mihret et al. **[37]	Y	N	Y	N	N	NA	Y	Y	N	5
**Oosthuizen et al. **[39]	Y	N	Y	Y	N	NA	Y	Y	Y	7
**Ratcliffe et al. **[40]	Y	Y	Y	N	N	NA	Y	N	N	5
**Ratcliffe et al. **[41]	Y	Y	Y	N	N	NA	Y	Y	N	6
**Umbeli et al. **[42]	Y	Y	Y	N	N	NA	Y	UC	N	5
**Total % of positive scores**	100	55	100	11	0	11	100	66	55	

*JBI critical appraisal checklist for quasi-experimental studies (Y* = *Yes, N* = *No, UC* = *Unclear N/A* = *Not Applicable)*

*Q1* = *Is it clear in the study what is the 'cause' and what is the 'effect' (i.e. there is no confusion about which variable comes first)?*

*Q2* = *Were the participants included in any comparisons similar?*

*Q3* = *Were the participants included in any comparisons receiving similar treatment/care, other than the exposure or intervention of interest?*

*Q4* = *Was there a control group?*

*Q5* = *Were there multiple measurements of the outcome both pre and post the intervention/exposure?*

*Q6* = *Was follow up complete and if not, were differences between groups in terms of their follow up adequately described and analyzed?*

*Q7* = *Were the outcomes of participants included in any comparisons measured in the same way?*

*Q8* = *Were outcomes measured in a reliable way?*

*Q9* = *Was appropriate statistical analysis used?*

**Table 3:** Critical appraisal results for the quasi-experimental studies included in the review

Studies	Appraisal questions													Score
	Q1	Q2	Q3	Q4	Q5	Q6	Q7	Q8	Q9	Q10	Q11	Q12	Q13	
**Kujawski et al. **[22]	Y	N	N	Y	N	UC	Y	NA	Y	Y	Y	Y	Y	9
**Montagu et al. **[38]	Y	N	N	UC	N	UC	Y	NA	Y	Y	Y	Y	Y	8
**Brown et al. **[36]	Y	N	N	UC	N	UC	N	NA	Y	Y	UC	N	N	4
**Total % of positive scores**	100	0	0	0	0	0	66	0	100	100	66	66	66	

*JBI critical appraisal checklist for randomised controlled trials (Y* = *Yes, N* = *No, UC* = *Unclear N/A* = *Not Applicable)*

*Q1* = *Was true randomization used for assignment of participants to treatment groups?*

*Q2* = *Was allocation to treatment groups concealed?*

*Q3* = *Were treatment groups similar at the baseline?*

*Q4* = *Were participants blind to treatment assignment?*

*Q5* = *Were those delivering treatment blind to treatment assignment?*

*Q6* = *Were outcomes assessors blind to treatment assignment?*

*Q7* = *Were treatments groups treated identically other than the intervention of interest?*

*Q8* = *Was follow up complete and if not, were differences between groups in terms of their follow up adequately described and analyzed?*

*Q9* = *Were participants analyzed in the groups to which they were randomized?*

*Q10* = *Were outcomes measured in the same way for treatment groups?*

*Q11* = *Were outcomes measured in a reliable way?*

*Q12* = *Was appropriate statistical analysis used?*

*Q13* = *Was the trial design appropriate, and any deviations from the standard RCT design (individual randomization, parallel groups) accounted for in the conduct and analysis of the trial?*

Multiple outcome measurements before and after the intervention were also identified when analysing the suitability of the included studies. If multiple surveys were conducted before and after the intervention, the authors would have ascertained whether the observed changes occurred naturally in the absence of the interventions or were due to the intervention they implemented. Because interventions were given at the cluster level (health facility level or community level) to different study participants before and after the implementation of interventions, participant follow-up was not achievable in all studies, except for the [35] study in which data were collected from the same participants before and after the intervention. All quasi-experimental studies which measured both before and after the intervention utilised the same measurement tools; however, the reliability and validity of the tools used to measure the outcome were not reported in three of the nine studies [35, 40, 42]. Although all studies performed analyses aimed to investigate specific effects of the interventions, the influences of other confounding factors were not controlled for in the statistical analyses of six of the nine quasi-experimental studies [35, 37, 39–42].

All three cluster RCT studies randomly assigned the intervention to the clusters, as shown in Table 3. As the interventions in these studies were held at the cluster level, it is less likely to expect concealment of the intervention assignment to the groups, and all of them were scored as not concealed [22, 36, 38]. Even though the groups were recruited by matching samples, significant socio-demographic variation between the survey respondents existed before and after the interventions in each group of all three studies.

Information regarding blinding in the studies was considered inadequate. Therefore, biases in both the performance of the intervention and detection of the outcome by the personnel (health care providers) and outcome assessors (data collectors in this instance) have the potential to be present. Only Kujawski et al. [22] described and acknowledged the possibility of information contamination among women before recruiting them to participate in the study (initial awareness regarding the presence of intervention at a specific health facility). Prior information regarding the presence of intervention may have influenced self-selection to the interventional facility. In the remaining two cluster RCTs [36, 38], there was no clear information regarding the level of bias introduced by knowledge of the intervention by participants (women) and outcome assessors. As the recruitment of women was performed after the intervention was implemented in all three studies, concealment of the participant groups to the personnel who delivered the intervention was not possible. Both the control and intervention groups were treated identically in Kujawski's [22] and Montagu's [38] trials, while additional unrelated interventions occurred in the control groups in Brown's [36] trial. While participants in all these studies were analysed in the groups in which they were initially randomised and the outcomes were measured similarly in both groups, the tools used to measure the outcome were not replicable, and no appropriate statistical analysis was conducted in one cluster trial [36].

### Interventions

Interventions varied between studies, ranging from health care provider RMC training to community engagement strategies for reducing mistreatment and policy amendments. This section describes the types of interventions by classifying them as multi-component, training-based, quality improvement, and companion of birth.

#### Multi-component interventions

Three pre- and post-studies [23, 40, 41] and one cluster RCT [22] study evaluated multi-component interventions targeting health care providers and/or women and community members. Abuya et al.'s [23] before and after study in Kenya evaluated the interventions designed to lower the rate of disrespectful and abusive behaviours under the Hashima project at the policy, community, health facility (13 facilities), and individual levels. They evaluated the effects of the interventions by surveying 1,369 women at discharge from the facility (641 women before and 728 women after intervention). In this study, RMC training was provided to health care providers and policymakers to enhance their understanding of the existence of disrespect and abusive care. Workshops on women's reproductive rights were also examined. The workshops were led by community members to enhance the relationships between community agencies and health facilities. Through technical meetings, the researchers also conducted continued policy dialogues with government representatives, professional associations, and civil society.

Similar to the results reported by Abuya et al.'s [23] study, Kujawski et al.'s. [22] cluster RCTs evaluated a two-stage intervention at the community and health facility levels. They designed a client service charter to create standards for mutual respect enacted by community and health-facility providers. The content of these community-level charters was then adapted by health facilities and incorporated into quality improvement activities. These quality improvement activities include ensuring privacy during admission and examinations, transparency of processes and care, trust-building mechanisms, and anonymous exit surveys to measure women's satisfaction. The effectiveness of the quality improvement activities developed from the client service charter was evaluated by surveys involving 1680 women (769 women in the control group and 1001 women in the intervention group) compared to the baseline surveys of 1388 women (744 women in the control group and 644 women in the intervention group) in the control and intervention groups.

In a pre- and post-study (70 woman at baseline and 149 women after intervention) conducted in Tanzania [40, 41], the authors evaluated two facility-based interventions which aimed to mitigate the mistreatment of women and enhance delivery of RMC. The intervention included an antenatal care education program for women in the third-trimester of pregnancy, drawing attention to the low information level among women as identified in a survey performed before designing the intervention [44]. They also implemented workshops with health care providers after examining the barriers and enablers to RMC. The evaluation assessed whether the workshops impacted women's experiences of disrespect and abuse by examining whether the survey results varied between the populations sampled before and after the interventions were introduced.

#### Training based intervention at facility levels

The common method of implementing change toward RMC at the health facility level is health care provider training/simulation and using pre- and post-intervention data for evaluation purposes. Three studies in Ethiopia [34, 35, 37] evaluated the effects of health care provider RMC training, followed by infrastructure improvements such as resource availability, visual prompts (posters), recognition of providers who adhere to RMC, and post-training health care provider supportive supervisions.

Other health care providers' training focusing on RMC incorporates information about creating awareness about women's experiences [39], ways of treating women with dignity and respect, communication, respecting women's autonomy, birth choices and preferences, and encouraging birth companions [33, 42]. Further health facility interventions in the included studies were a quality improvement workshop aimed at improving health providers' ability to deliver RMC through a plan-do-study-act cycle [38].

#### Birth companion

Another means of augmenting RMC included the presence of a birth companion for labouring women. A cluster RCT study from South Africa [36] evaluated the benefits of promotive strategies toward the availability of birth companions. They encouraged the uptake of birth companions by providing educational intervention to promote childbirth companions in interventional hospitals compared to usual care in control hospitals [36].

### Effectiveness of interventions

The effect measures of the outcomes of the studies are described in the summary of findings table (Table 4). We synthesised the effectiveness of interventions by grouping studies in the outcomes the programs’ focused on achieving and the categories of interventions implemented. We reviewed the reduction of mistreatment during maternity care and a change in respectful maternity care, women's satisfaction, health care provider perceptions of RMC, and the employment of birth companions for women during labour and birth. Changes in these outcomes are presented in the following sub-sections.

**Table 4:** Summary of findings table

Multi-component RMC intervention compared to usual care for reduction of mistreatment of women and/or enhancing RMC			
**Population**: Healthy women during maternal health care utilisations **Setting**: Maternity care units (antenatal, labour and postnatal wards) in Ethiopia, Sudan, Kenya, Tanzania, South Africa, Ghana & India **Intervention**: Multi-component RMC intervention **Comparison**: usual care Data sources: all data sources were from women self-report in primary studies— some effect measures were calculated based on summary findings by reviewers if not given in primary articles			
**Outcomes**	**Anticipated absolute effects** Single effect size was not pooled, and narrative synthesis of each studies effect measures were given	№ of participants (studies)	Certainty of the evidence (GRADE)
**Any form of mistreatment of women**	The effect measure from one cluster RCT [22] — aOR = 0.34, 95% CI: (0.21–0.58) (3.2% in intervention groups vs 15.76% in control groups). Observation studies: four pre- and post-studies showed reduction in any forms of mistreatment; aOR = 0.58, 95% CI: ( 0.43, 0.79) (20% before vs 13.2% after intervention) [23], 18% risk reduction— aIRR = 0.82( 95% CI 0.74, 0.91) [34]. Other two pre-post studies showed absolute risk reduction from crude OR of 0.07(95% CI 0.05—0.1) (71.8% vs 15.9%) [37] and OR = 0.08, 95% CI: 0.043—0.17) (18% after intervention vs 70% before intervention) [41]	8680 (5 studies—1 RCT [22] and 4 pre-and-post observational studies [23, 34, 37, 41])	⨁⨁⨁◯ Moderate^a,b^
**Physical abuse**	Effect measures from two cluster RCTs: [22] — (OR: 0.22, 95% CI: 0.05–0.97) (1.8% control vs 0.3% intervention), while the other cluster RCT did not report summary statics but identified it as significant [36]. Pre-and-post studies also showed reduction of physical abuse— (OR 0.5; 95% CI: 0.3–0.9) (4.2% before vs 2.1% after intervention) [23], absolute risk reduction from 16.7% before to 8. 9% after intervention [34], 61% at baseline to 15.4% at the postintervention [37] and from 52 to 1% [41]	7830 (6 studies— 2 cluster RCTs [22, 36] and 4 pre-and-post observational studies [23, 34, 37, 41])	⨁⨁◯◯ Low^a^
**Verbal abuse**	Verbal abuses were decreased with effect measures from pre-and-post studies— (OR 0.6; 95% CI: 0.4–0.8) (18.0% before vs 11.3% after intervention) [23], decreased from 78% to 24.4% [37], 54% to 5% [41]. Even though not significant, non-dignified care was also decreased in one cluster RCT [22] 2.2% vs 11.2% (aOR = 0.58, 95%CI: 0.3–1.1) and in another pre-and-post study by [34]— from 8.6% to 5.8%	5772 (5 studies— 4 pre-and-post observational studies [23, 37, 41] [34] & 1 cluster RCT [22])	⨁⨁⨁◯ Moderate^a^
**Non-confidential care**	Effect measures for decrement of non-confidential care— (OR 0.5; 95% CI: 0.2–0.9) (3.9% before vs 1.8% after intervention) [23], 69% reduced risk, RR = 0.31, 95% CI: (0.26—0.37) (79.5% vs 24.7%) [37] and 98% reduced risk, RR = 0.02 95% CI: (0.01—0.10) (54% vs 1%) [41]	2326 (3 pre-and-post observational studies [23, 37, 41])	⨁⨁◯◯ Low^c^
**Non-consented care**	Effect measures —18% absolute risk reduction (83.3% vs 65.3%, RR = 0.78, 595% CI: (0.70—0.90) [34]. Similarly, Mihret et al. [37] showed 52.9% risk reduction (69.9% vs 17.1%, RR = 0.24, 95% CI: (0.19—0.31) while [41] showed 4% risk reduction from 5 to 1%. Unlike these three studies, Abuya's [23] pre-and-post study highlighted increment of the risk after intervention by 20% (61% vs 81%; aOR = 3.4, 95%CI: 2.5–4.7)	2545 (4 pre-and-post observational studies [23, 34, 37, 41])	⨁⨁◯◯ Low^c^
**Privacy violated**	From pre-and-post study- effect measures showed decrement of privacy violation—52.5% reduction (79.7% to 27.1%; RR = 0.34, 95%CI: (0.28—0.41) [37] and [41]—50.4% reduction (53.1% vs 2.7%; RR = 0.05, 95%CI: (0.02—0.13). Although not significant additional two pre-and-post study also showed reduction in privacy violation—from 7.4% to 5.7%; aOR = 0.69, 95% CI: (0.44 – 1.08) [23] and from 81.8% to 77.4%, RR = 0.95, 95%CI: (0.85 —1.05) [35]	2708 (4 pre-and-post observational studies [23, 35, 37, 41].)	⨁⨁◯◯ Low^c^
**Respectful maternity care**	Two cluster RCTs showed an increment of RMC:—person-centred maternity care score raised by 22.9 points (95%CI: 20.9—25.0) [38] and — aRR 3.44 (2.45—4.84) [22]. Similarly, four pre-and-post studies also showed improvement of RMC related to intervention—18 points increment in RMC: (β = 17.6, 95% CI: (15.6—19.6), a relative increase of 43% from 50 to 72 [33]; increased by 36.4% (38.1% to 74.5%) [39]; respectful care increased from none at baseline to 22.8% at the time of evaluation [40] and 5% increment in RMC (89.7% vs 94.7%) [42]	13,119 (6 studies— 2 cluster RCTs [22, 38] and 4 pre-and-post observational studies [33, 39, 40, 42])	⨁⨁⨁◯ Moderate^a^

High certainty: we are very confident that the true effect lies close to that of the estimate of the effect

Moderate certainty: we are moderately confident in the effect estimate: the true effect is likely to be close to the estimate of the effect, but there is a possibility that it is substantially different

Low certainty: our confidence in the effect estimate is limited: the true effect may be substantially different from the estimate of the effect

Very low certainty: we have very little confidence in the effect estimate: the true effect is likely to be substantially different from the estimate of effect

*aOR* adjusted odds ratios, *CI* confidence interval, *IRR* incidence risk ratio, *RMC* respectful maternity care, *RCT* randomised controlled trial

**Explanations**

^a^The cluster RCT was assessed as having a serious risk of bias due to lack of allocation concealment, blinding, and variation in patient characteristics and patient-reported outcomes. All observational studies were also assessed as having a serious risk of bias because of lack of allocation concealment, blinding, randomization, patient-reported outcome, failure to adequately control for potential confounders in two studies, and lack of repeated measurement both before and after the intervention

^b^The inconsistency between the studies could be due to the observed variations in the implemented intervention and outcome measurements

^c^The findings were based only on observational studies that had a serious risk of bias because of lack of allocation concealment, blinding, randomization, patient-reported outcome, failure to adequately control for potential confounders in two studies, and lack of repeated measurement both before and after the intervention in all studies. Additionally, the data were based on patient/women/ reports

#### Mistreatment of women in health facilities

Moderate certainty evidence emerged from two cluster RCTs [22, 36] and four pre- and post-studies [23, 34, 37, 41] assessed the effects of multi-component interventions in diminishing mistreatment of women as reported by women in health facilities.

Kujawski et al. [22] projected implementing client service charters at the community and health facility level was associated with reduced odds of a woman experiencing mistreatment of women by two-thirds (aOR = 0.34, 95% CI: 0.21–0.58, 2983 participants) as compared to control groups. Other pre-post interventional studies [23, 41] also reported the possibility of reducing the odds of mistreatment of women through multi-component interventions. Abuya et al. [23] revealed that multi-component interventions were associated with a reduction in the odds of women experiencing humiliation and disrespect by 42 per cent (aOR = 0.58, 95% CI: 0.43–0.79, absolute risk reduction = 7%, 1369 participants). Ratcliffe et al. [41] also revealed the benefits of implementing interventions on health care providers and women. They reported that the prevalence of disrespect and abuse experienced by women was reduced from 70 per cent at baseline to 18 per cent after intervention (absolute risk reduction = 52%, 219 participants).

Other pre- and post-intervention studies have also shown a declining trend in women experiencing mistreatment in maternity care after implementation of interventions [34, 37]. Asefa et al. [34] reported the number of mistreatment categories that women experienced was declined by 18 per cent after implementation of RMC promotive interventions through training and supportive supervision (adjusted exponent of β = 0.82, 95% CI: 0.74–0.91, 388 participants). Mihret et al. [37] study, implementing health care provider training, concluded that the prevalence of any mistreatment that women experienced was reduced from 71.8 per cent in the pre-intervention group to 15.9 per cent in the post-intervention group (absolute risk reduction = 56%, 738 participants).

One cluster RCT [36], with significant methodological weaknesses, evaluated the effects of an intervention to promote the importance of a birth companion being present. Consistent with methodologically flawed research, their findings were insignificant. While the authors did not perform appropriate statistical analysis to estimate an intervention’s effect size, physical abuse was reported to be reduced from 2 per cent to 1 per cent in the intervention arm. In comparison, it increased from 3 per cent to 4 per cent in the control group. Although the difference was not statistically significant, the risks associated with other types of mistreatments, such as verbal abuse and abandonment of care, increased in both arms of the RCT, as opposed to physical abuse. Verbal abuse (being shouted at) and abandonment (being left alone) increased from 14 per cent and 12 per cent before intervention levels to 15 per cent and 16 per cent after intervention in interventional groups (4148 participants).

#### Respectful maternity care

Moderate certainty evidence from two cluster RCTs [22, 38] and four pre- and post-interventional studies [33, 35, 39, 40, 42] suggests an enhancement of respectful maternity care secondary to various interventions in the community, health system, and facility levels (overall 13,119 study participants). Following the implementation of health care provider team-based quality improvement activities through plan-do-act-study cycles, Montagu et al. [38] reported an improvement in RMC scores (expressed as person-centred maternity care) by 22.9 points (95% CI: 20.9–25.0) in intervention groups compared to control group (1170 samples). Furthermore, Kujawski et al. [22] identified that implementation of community and health facility-based interventions was associated with increased respectful care from health care providers to women during their stay at the birth facility (RR: 3.44, 95% CI: 2.45–4.84, *p*-values < 0.0001, 2983 participants).

Similarly, a pre- and post-study by Afulani et al. [33] reported a relative increment of mean RMC (person-centred maternity care) score by 43 per cent from 50 per cent at baseline to 72 per cent after intervention implementation. While controlling for potential confounders, the RMC score after the intervention was 18 times higher than the baseline score (*β* = 17.6; 95% CI: 15.6‐19.6, 538 participants). Such increments were also observed in individual subscales for dignity and respect, communication and autonomy, and supportive care, with risk differences of 15, 87, and 55 per cent, respectively.

Based on a pre- and post-study, Oosthuizenez et al. [39] reported positive childbirth experiences (RMC) increased from 38.1 per cent at baseline to 74.5 per cent during follow-up in intervention groups (aOR = 4.33, p-value < 0.0001, 1332 participants). Umbeli et al. [42] evaluated the effects of training health care providers on communication skills to improve specific aspects of RMC. The proportion of women reporting perceived supportive, friendly, and respectful care from health care providers increased by 5 per cent (89.7% before training to 94.7 per cent after training, 4469 participants). Another pre- and post-study evaluated the effects of educating women on birth preparedness and complication readiness and training health care providers to mitigate mistreatment of women reported an increment of perceived respectful care to 22.8per cent compared to none at baseline (219 participants) [40].

Asefa et al. [35] assessed the positive outcomes of training health care providers on respectful maternity care. They concluded that training alone could only result in a minimum resolution of the mistreatment of women (i.e., lack of RMC). Nevertheless, they did report the proportion of health care providers who positively perceived RMC domains increased from 21.9 per cent before the training to 35.9 per cent after the training (*p*-value = 0.08, 64 participants).

#### Maternal satisfaction

Three pre- and post-studies [39, 40, 42] and one cluster RCT [22] evaluated whether interventions increased women's satisfaction with maternity care. These studies reported improved satisfaction after implementing interventions to address mistreatment during maternity care, however, there were limitations in the measurement of women’s satisfaction.. One of these studies, conducted by Ratcliffe et al. [40], reported a significant increase in maternal satisfaction from 12.9 per cent before the intervention to 75.8per cent after training health care providers, suggesting that effective interventions can lead to improved satisfaction despite the limitations in measurement (219 participants). Umbeli et al. [42] and Oosthuizen et al. [39] also suggested an improvement in maternal satisfaction by 5.9 per cent (89.8% at baseline to 95.7% after the intervention, 4469 participants) and 26.6 per cent (47.0% at baseline to 73.6% post-intervention, 1332 sample), respectively. However, Kujawski et al.'s [22] cluster RCT showed little or no difference (aOR = 0.98, CI:0.91–1.06, *p*-value = 0.67, 2983 participants) between the intervention and control groups related to the proportion of women reporting being satisfied with the care provided.

## Discussion

This systematic review illustrates how community-, policy-, health system-, and health facility-level interventions can influence women's perceived experiences of mistreatment and/or respectful care during their maternal care encounters in health facilities. All papers included in the review implemented and evaluated various interventions which extended from quality improvement activities in health facilities to the community- and policy-inclusive strategic activities. Even though their effect sizes varied from study to study, most of the wide-ranging interventions were reported to have had made a positive effect in reducing the mistreatment of women and/or enhancing respectful maternity care in health facilities.

Downe et al. [24] published a systematic review that explored the roles of RMC policies in changing the intrapartum experiences of women, suggesting that such interventions could reduce non-respectful behaviours and practices in maternity care. In addition, Dhakal et al. [25] explored low-level evidence highlighting educational interventions and their effectiveness in promoting respectful maternity care. As such, this review builds on this evidence, focusing on multi-component interventions during the continuum of maternity care rather than focusing on specific components of interventions, thus highlighting the complexity of the phenomenon of mistreatment.

Given the complex nature of mistreatment of women, the reviewed studies suggest that interventions targeting all system approaches (health facilities, communities, health systems, and policies) are required to be executed in order to bring about sustained and transformational change for women. This could be achieved by improving interpersonal relationships between HCPs and women, addressing health facility and health system constraints such as shortage of skilled staff inadequate medical supplies, and implementing policies that empower community engagement in health care decisions. Even though such interventions echo the complexity of the issue and are therefore heterogeneous, and it is difficult to quantitatively ascertain their cumulative effects, the clinical significance of such interventions are indispensable in addressing the complex drivers of the mistreatment of women. The importance of initiating a multi-factorial response to the phenomenon of mistreatment and the varied characteristics of maternity care settings across the globe has been established in the *Bangkok Charter for health promotion* and *WHO intrapartum recommendations* [29, 45].

Our review identified that the overall experience of mistreatment of women was reduced by at least half when related to multi-component interventions in pre-post and cluster RCT studies [22, 23, 41]. Individual interventions enable a shift in knowledge and attitudes that allow behavioural change and mutual respect between HCPs and clients. Changing individual HCPs' and clients' behaviours cannot be sustained without improving both health facilities and the overall health system by enacting decisive policy actions by engaging with communities and users of the service. Designing and implementing impactful strategies aimed at reducing mistreatment and enhancing RMC for women must address the unique and hierarchical levels of health facilities, including cultural and institutional change. Including higher-level interventions would not only sustain positive interaction between health care providers and women but would also address the higher-level drivers of mistreatment of women beyond individual behaviours. Although the benefits of interventions that engage communities in health care interventions were well established and frequently advocated in previous studies [46–48], only four of the studies reviewed engaged community members in the development of their interventions. This may reflect the challenges women and families face when receiving disrespectful maternity care and the obstacles they face as a result of diminished agency when advocating for the delivery of RMC in an inhospitable health setting. However, engaging communities in future interventions still remains necessary to achieve a sustainable reduction in the mistreatment of women and to reduce the normalisation of women's mistreatment during maternity care.

Multi-component interventions implemented at various levels within an organisation did not clearly inform which set of interventions successfully addressed the intended goal for quality and respectful care. Specific interventions that either targeted enhancing health care providers' awareness of respectful care or quality improvement activities were seen to reduce the mistreatment of women [34, 37]. Staff attitude and value transformation training were also found to reduce mistreatment of women; however, the overall success in minimising abusive care appeared to be more significant when the interventions were directed at changing the health facility's circumstances by providing the facility and staff with essential supplies, drugs, and equipment. Recognising health care providers' efforts to provide respectful care is also crucial. Although an impossibility for many health settings, this could be realised through motivational strategies and counselling services for health providers, the provision of greater staffing and equipment resources, supporting them in managing high workloads, critical incidents, and trauma, as identified in Mihret et al. [37] and Abuya et al. [23]. Recognising the daily struggles of health care providers in health facilities, including coping with resource limitations, underpayment, and high patient load, can enhance their sense of value and respect in their role.

The presence of a birth companion has been shown to be crucial in addressing inequalities, improving emotional support, and maternal and newborn health outcomes [49]. It is also highlighted as a critical component of respectful maternity care by the World Health Organization [50]. This review’s findings further support the significance of having a birth companion; while there is no consistency of effect in reducing all forms of mistreatment of women, Brown et al. [36] showed a reduction in physical abuse when a birth companion was present. The authors indicated that the implementation of birth companions was more challenging than expected, especially in health care systems with limited resources and frequent turnover of staff [36]. To better advocate and implement interventions to routinely incorporate birth companionship, it is essential to address all factors that may hinder the positive outcome of having a birth companion [51]. This includes a health facility-level commitment to enacting the policy and facilitating the physical space required for a birth companion to be present without compromising a woman's privacy.

Mistreatment and RMC are not necessarily two direct sides of the same coin. Reducing or eliminating women's mistreatment does not necessarily mean that respectful maternity care is present within a facility. Any effort to improve the quality of care should uphold the safety and dignity of women by situating respectful maternity care at the core of its service [21]. Accordingly, research which focused on quality improvement activities were included in the review. Quality improvement activities based on local health facilities' attempts to manage the number of women accessing the service and strategies to avoid or manage the busyness of the facility were included. Such interventions enable health facilities to assess adaptable changes through continuous plan-do-study-acts (PDSA) [38]. Likewise, scenario-based integrated simulation training for health care providers was highlighted as an intervention that enhanced woman-centred maternity care through the change of behaviours in practice [33]. Such training can allow health care providers to learn, practice, and reflect on stressful situations and minimise spontaneous reactions which can be abusive to women. If such interventions also include efforts to change infrastructure limitations and motivational strategies for HCPs, they could bring about sustainable changes in respectful maternity care.

The importance of good rapport between a health care provider and a woman is vital in building mutual respect and understanding [52, 53]. However, most of the studies included in this review overlooked the importance of this factor. Only Umbeli et al. [42] explored this concept, revealing that improved communication skills with HCPs can increase women's respectful care in a supportive and friendly manner. However, equal attention should be given to reducing and eliminating all forms of mistreatment, enhancing communication, and achieving respectful care is difficult to realise in a facility where mistreatment is normalised.

It must be noted that this review has some limitations that we have considered. First, it includes studies that differed in study design and methods and studies that measured both the mistreatment of women and respectful maternity care. This reflects the complexity of the phenomenon across each health care setting, but in this review contributed to the heterogeneity of the studies and prevented pooling of intervention effect sizes in the meta-analysis. The lack of a meta-analysis does not limit the quality of this review. We performed the synthesis without meta-analysis by categorising intervention effects based on the types of studies, interventions implemented, and quality of the studies, as recommended by Campbell et al. [43]. Second, we only included studies and papers written in English. Therefore, there is a possibility that we may have missed some important reviews written in other languages; however, we believe the study selection bias due to this inclusion criteria are minimal, as most major high-quality peer-reviewed journals are published in English, reflecting the research community’s desire to reach a wide audience [54].

## Conclusions

Interventions aimed at reducing women’s mistreatment and enhancing respectful maternity care should simultaneously incorporate multiple and varied approaches in order to positively affect women’s clinical experiences during childbearing. Low to moderate certainty evidence suggests that multi-component interventions were effective in reducing the mistreatment of women and/or enhancing respectful maternity care in health facilities. Interventions that motivate health care providers through various recognition strategies were found to be more successful in bringing change than interventions that only focused on training at an individual level. Future interventions should consider incorporating individuals, health facilities, health systems, policy-level drivers, and community/consumer engagement in the mistreatment of women and the determinants of respectful care.

## Supplementary Information

**Additional file 1.** 

**Additional file 2.**

## Data Availability

The dataset generated from primary articles and/or analysed during the study are available from the corresponding author on reasonable request.

## Abbreviations

AOR: Adjusted odds ratio
CI: Confidence interval
GRADE: The Grading of Recommendations, Assessment, Development, and Evaluation
HCP: Health care providers
IRR: Incidence risk ratio
JBI SUMARI: JBI System for the Unified Management, Assessment, and Review of Information
PDSA: Plan-do-study-acts
PICO: Population, Intervention, Comparator and Outcomes
PRISMA: Preferred Reporting Items for Systematic Reviews and Meta-Analysis
PROSPERO: Prospective Register of Systematic Reviews
RCT: Randomized controlled trials
RMC: Respectful maternity care
SSA: Sub-Saharan African
WHO: World Health Organization
