# Supplementary material for: The roles of multi-component interventions in reducing mistreatment of women and enhancing respectful maternity care: a systematic review
Source: BMC Pregnancy Childbirth. 2023 May 1;23:305. doi: 10.1186/s12884-023-05640-3 (PMC10150509; doi:10.1186/s12884-023-05640-3)
Supplement: Supplementary file 2 — Additional file 2. [file 12884_2023_5640_MOESM2_ESM.docx]

**Additional file 2: Database search strategies**

| **Databases** | **Search terms** | **Date searched** | **Search hits** |
| --- | --- | --- | --- |
| PubMed | (((((((((((((((maternity care) OR (antenatal care)) OR (labor)) OR (labour)) OR (birth)) OR (childbirth)) OR (intrapartum)) OR (postpartum[MeSH Terms])) OR (postnatal)) OR (puerperium[MeSH Terms])) OR (puerperium)) AND ((((((intervention*) OR (intervention[MeSH Terms])) OR (policy)) OR (intervention)) OR (program)) OR (strategy))) AND (((((((respectful maternity care[MeSH Terms]) OR (respectful maternity care)) OR (respect)) OR (dignified)) OR (confidential)) OR (respect*)) OR (humanized))) AND ((((((disrespect and abuse[MeSH Terms]) OR (disrespect and abuse)) OR (mistreatment)) OR (violence)) OR (obstetric violence[MeSH Terms])) OR (dehumanized))) AND (((((women[MeSH Terms]) OR (women)) OR (maternal)) OR (mothers)) OR (maternal[MeSH Terms]))) AND (((evaluation) OR (assessment)) OR (appraisal)) | Dec 13 2021 | 456 |
| CINAHL | (matern* OR prenatal care OR antenatal care OR labor OR labour OR birth OR childbirth OR intarnatal OR parturition OR postnatal OR postpartum OR puerperium) AND (intervention* OR polic* OR program* OR strateg*) AND (respectful maternity care OR respect* OR digni* OR confidential OR humanised OR humanized) AND (disrespect and abuse) OR mistreatment* OR disrespect* OR abus* OR violence OR obstetric violence OR dehumani* OR non dignified OR discrimination OR neglect OR abandonment) AND (women OR maternal OR mother* ) AND (evaluation* OR assessment* OR appraisal*) | 10^th^ Nov 2021 | 454 |
| Ovid Embase | ((maternity or maternal or prenatal care or antenatal care or labor or labour or birth or childbirth or intranatal or parturition or postnatal or postpartum or puerperium) and (intervention* or policy or program* or strategy) and (respectful maternity care or respect* or dignity or confidential or humanised or humanized) and ((disrespect and abuse) or mistreatment* or disrespect* or abuse or abuse or violence or obstetric violence or dehumanized or dehumanised or non-dignified or discrimination or neglect or abandonment) and (women or maternal or mother*) and (evaluation* or assessment* or appraisal*)).af. | 17^th^ Dec 2021 | 298 |
| Scopus | (respectful AND maternity AND care OR respect OR dignified OR confidential OR humanized) AND (mistreatment OR disrespect OR abuse OR obstetric AND violence OR dehumanized) AND (maternity AND care OR antenatal AND care OR labour OR birth OR intrapartum OR postnatal) AND (intervention OR policy OR program OR strategy) AND (women OR maternal OR mothers) | 13^th^ Dec 2021 | 171 |
